# Reduced mechanical shear is associated with early cortical differentiation and GDNF-GFRA1/RET signalling in human brain organoids

**DOI:** 10.1093/stcltm/szag070

**Published:** 2026-09-08

**Authors:** Zizhen Zhao, Jingran Du, Shan Zhang, Haowen Shi, Hui Chen, Hong Cheng, Lihua Zhou, Chenju Yi, Jiachuan Wang

**Affiliations:** Shenzhen Traditional Chinese Medicine Hospital, Affiliated to Nanjing University of Chinese Medicine, Shenzhen, 518033, China; Department of Geriatrics, The Fourth Clinical Medical College of Guangzhou University of Chinese Medicine, Shenzhen Traditional Chinese Medicine Hospital, Shenzhen, 518033, China; Department of Obstetrics and Gynecology, The Seventh Affiliated Hospital of Sun Yat-sen University, Shenzhen, 518107, China; Yangzhou Maternal and Child Health Care Hospital Affiliated to Yangzhou University, Yangzhou, 225006, China; Department of Anatomy, School of Medicine (Shenzhen), Sun Yat-sen University, Shenzhen, 518107, China; School of Life Sciences, Faculty of Science, University of Technology Sydney, Ultimo, NSW, 2007, Australia; Department of Geriatrics, The Fourth Clinical Medical College of Guangzhou University of Chinese Medicine, Shenzhen Traditional Chinese Medicine Hospital, Shenzhen, 518033, China; Department of Anatomy, School of Medicine (Shenzhen), Sun Yat-sen University, Shenzhen, 518107, China; Department of Geriatrics, The Seventh Affiliated Hospital of Sun Yat-sen University, Shenzhen, 518107, China; Guangdong Provincial Key Laboratory of Digestive Cancer Research, Shenzhen, 518107, China; Shenzhen Key Laboratory of Chinese Medicine Active Substance Screening and Translational Research, Shenzhen, 518107, China; Shenzhen Traditional Chinese Medicine Hospital, Affiliated to Nanjing University of Chinese Medicine, Shenzhen, 518033, China; Department of Geriatrics, The Fourth Clinical Medical College of Guangzhou University of Chinese Medicine, Shenzhen Traditional Chinese Medicine Hospital, Shenzhen, 518033, China

**Keywords:** brain organoid, low-shear culture, early cortical differentiation, GDNF receptor, neuroepithelial expansion

## Abstract

Early cortical differentiation in human brain organoids is a critical step of corticogenesis, but remains poorly coordinated under conventional culture conditions. In a standard orbital shaker system, mechanical shear and variability in extracellular matrix support, can disrupt neuroepithelial organisation, leading to inconsistent early cortical differentiation. Here, we investigated how ClinoStar-associated low-shear culture conditions affect early corticogenic marker expression in brain organoids and examined GFRA1/RET-related signaling as a candidate pathway associated with these culture-related effects. Using a low-shear ClinoStar bioreactor, we observed improved neuroepithelial-like expansion, enhanced tissue organisation, and promoted early cortical differentiation during the neuroepithelial expansion stage. Compared with conventional conditions, low-mechanical shear in ClinoStar was associated with increased proliferation capacity and supported the number of TBR2^+^ intermediate progenitors and TBR1^+^ early cortical neurons. Transcriptomic analysis revealed enrichment of neuronal differentiation and neuroactive ligand-receptor interaction pathways, with increased upregulation of GDNF signalling components under reduced mechanical shear. Functional experiments showed that GDNF supplementation increased TBR2^+^ intermediate progenitor-associated and TBR1^+^ early neuronal marker-related populations in brain organoids, suggesting a positive effect on early corticogenic marker expression. Consistent with this, GFRA1/RET levels were elevated in organoids in the ClinoStar culture with low-mechanical shear stress, and manipulation of GFRA1 affected early cortical differentiation outcomes. These findings suggest that ClinoStar low-shear culture promoted early corticogenic marker expression, potentially associated with GDNF-GFRA1/RET signalling. Collectively, this study provides a refined culture framework for improving the reproducibility and structural organisation of human brain organoid models of early cortical differentiation.

## Graphical Abstract

**Graphical Abstract:**
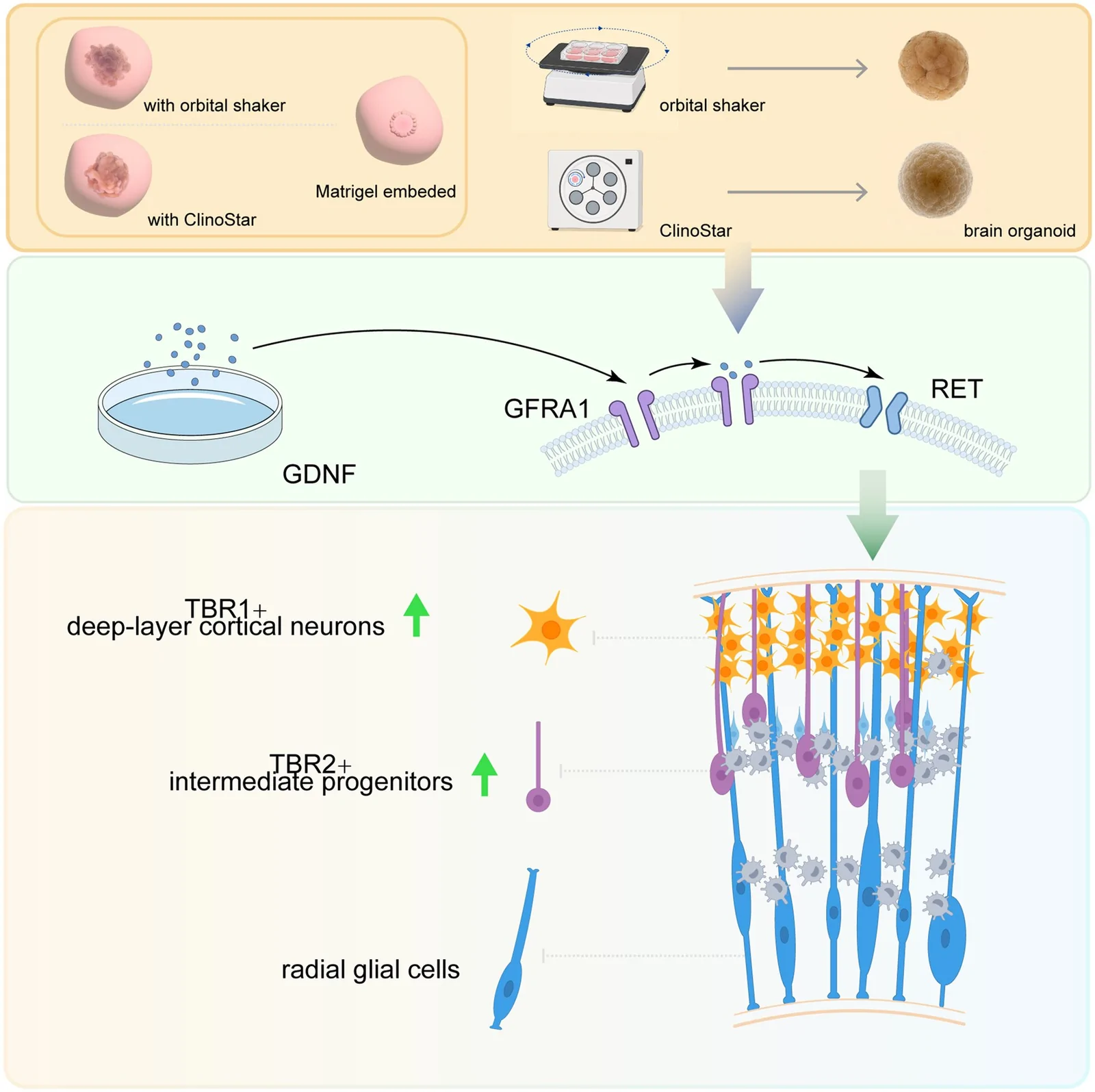

Significance Statement Human brain organoids are valuable models for studying early brain development, but their structure and differentiation often vary under standard culture conditions. This study shows that a low-shear rotational culture platform improves organoid organization and supports early cortical differentiation. It also identifies neurotrophic factor-related signaling as a potential contributor to these culture-associated effects. These findings provide a practical approach for improving the consistency and developmental quality of brain organoid models, which may benefit studies of human brain development, disease mechanisms, and future therapeutic applications.

## Introduction

The concept of human brain organoids (hBOs), 3-dimensional, self-organizing neural tissues, derived from pluripotent stem cells (PSC), was first introduced in 2013 with the landmark publication by Lancaster and colleagues,^1^ who described the generation of hBOs that recapitulate key features of early human brain development. This groundbreaking work enabled researchers to model neurodevelopmental processes and disorders in vitro, providing a powerful platform for studying aspects of human-specific brain development that was unable to be studied in animal models. Since their introduction, hBOs have revolutionized neuroscience research by offering unique benefits, including the ability to model patient-specific conditions, test therapeutic interventions, and explore lineage transitions and cellular interactions within a controlled environment, greatly advancing our understanding of human brain biology.

Since the introduction of hBOs technology in 2013, there have been continuous refinements and developments of new culture methods to improve the structural and functional fidelity of these models. Innovations include optimizing bioreactor systems to reduce mechanical stress, improving extracellular matrix (ECM) compositions, and incorporating defined neurotrophic factors to better represent in vivo conditions. These advances have significantly enhanced the reproducibility and organization of early cortical structures in vitro. Current hBOs methods can generate ventricular zone-like structures containing SOX2^+^ radial progenitor-like cells, together with cells expressing cortical projection neuron layer-associated transcription factors, such as SATB and CTIP2.^2^ However, many yielded hBOs still lack fully organized cortical layers, with inconsistent transition from subventricular zone-like intermediate progenitor cells (TBR2^+^) to early-born deep-layer neurons (TBR1^+^).^3^ As a result, they often fail to replicate the sequential architecture of human corticogenesis,^4^^,^^5^ limiting their accuracy in modeling early cortical development.

The above major limitation is because organoid development is highly sensitive to mechanical forces during culture. Standard orbital shaker systems subject organoids to uneven shear stress and turbulent fluid dynamics, which can disrupt apical-basal polarity, weaken rosette stability, and hinder the formation of distinct cortical layers.^6^ Studies have shown that excessive shear stress introduces mechanical instability, resulting in fragmented or flattened neuroepithelial surfaces and reduced formation of ventricular-like zones.^7^ Additionally, unregulated shear force affects local oxygen and nutrient gradients, causing metabolic stress that undermines cell viability and leads to inconsistent cell distribution within the organoid.^8^^,^^9^ A recent study using rotating chamber culture systems also showed that controlled fluid dynamics can minimize fluid-flow induced shear stress, thereby improving the reproducibility of hBO morphology.^8^ Furthermore, various ECM components are used in hBO culture, with Matrigel being the most commonly used. However, Matrigel is derived from Engelbreth-Holm-Swarm mouse sarcomas, and therefore the composition remains undefined and difficult to standardize across batches.^10^ Its variable biochemical and mechanical properties can alter neuroepithelial polarity, disrupt rosette morphology, and interfere with the establishment of structured cortical-like layers.^11^ In addition, differences in ECM stiffness or uneven matrix embedding may restrict tissue expansion, potentially distort apical-basal organization of the neuroepithelium, and result in mechanical stress within the tissue,^12^ compromising neuroepithelial integrity and resulting in inconsistent early-stage cortical fate specification in hBOs.

Neurotrophic factors such as Brain-derived neurotrophic factor (BDNF) and glial cell line-derived neurotrophic factor (GDNF) are essential regulators of neuronal differentiation, survival, and cortical maturation.^13^^,^^14^ BDNF primarily signals through the TrkB receptor encoded by NTRK2, while p75NTR encoded by nerve growth factor receptor (NGFR) is a low-affinity neurotrophin receptor that can modulate neurotrophin-related signaling.^15^ These receptors activate canonical downstream pathways, including MAPK/ERK, PI3K/AKT, and PLCγ, thereby promoting neurogenesis, neuronal survival, and dendritic maturation. In contrast, GDNF signals through the RET receptor in complex with GFRA1/2/3 co-receptors, and has been shown to regulate progenitor proliferation and the differentiation of cortical lineages.^16^ Both factors are often incorporated into hBO culture protocols, particularly during the maturation phase, to enhance neuronal growth, functional development, and synaptic formation.^17^

In this study, we focused on the neuroepithelial expansion stage, a critical period of rapid organoid growth and neuroepithelial rosette formation during early corticogenesis. We observed that this lineage progression is highly sensitive to culture-associated mechanical and matrix perturbations. Compared with conventional orbital-shaker culture, the ClinoStar bioreactor has a low-shear condition and yielded hBOs with better neuroepithelial integrity and significant changes in TBR2^+^ and TBR1^+^ populations suggestive of progenitor cell—neuron transition, while Matrigel embedding impaired this transition. To identify molecular mechanisms, we profiled transcriptional responses under different culture conditions and found that ClinoStar culture was associated with higher levels of GDNF receptor components (GFRA1/RET), suggesting enhanced GDNF signaling during the early differentiation window. Then, we further found that GDNF supplementation increased TBR2^+^ and TBR1^+^ marker-positive populations, suggesting a supportive effect on early corticogenic differentiation. Together, these experiments identify reduced mechanical shear as a permissive culture condition associated with enhanced GDNF responsiveness and improved early cortical differentiation, providing a framework to improve the reproducibility and organization of early cortical tissue in human hBOs.

## Material and methods

### hiPSC culture

hiPSCs (Nuwacell, Cat# RC1001; Research Resource Identifiers [RRID]: CVCL_A8HA) were maintained in Tesr1 medium. Vitronectin (Stemcell Technologies, Cat#07180) was diluted 1:24 in DPBS and coated at 37 °C for at least 1 h before seeding cells. When the centers of colonies began to darken and the space between colonies narrowed, the cells were separated using the non-enzymatic reagent ReleSR (Stemcell Technologies, Cat#100-0483) before the passage. After each passage, Y-27632 (Stemcell Technologies, Cat#72302) was added to the culture medium for cell activation on the first day, followed by regular Tesr1 medium for subsequent changes. Following each passage, hiPSCs were preserved using Cryostor CS10 (Stemcell Technologies, Cat#07930).

### Human brain organoid culture

Only hiPSCs from passages 10 to 15 were used for hBOs formation. When the hiPSCs reached 75% confluence in T75 flasks, the cells were dissociated using Gentle Cell Dissociation Reagent (Stemcell Technologies, Cat#100-0485), followed by centrifugation at 1000 rpm for 5 min. The cells were resuspended in Tesr1 medium (Stemcell Technologies, Cat#85850) containing Y-27632, and the cell density was adjusted to 10 000 cells/100 μL. A 96-well ultra-low attachment plate was used, with 100 μL of the cell suspension added to each well, where they self-assembled into embryoid bodies (EBs) within the first 24 h on an orbital shaker (DLAB, Cat#SK-O180-C) at 60 rpm. On days 2 and 4, 100 μL of Tesr1 complete medium was added. On day 5, smooth-edged embryoid bodies were transferred to a 24-well ultra-low attachment plate. The medium was replaced with neural induction medium, and the plate was shaken at 75 rpm for 3 days. After the neuroepithelial layer was properly formed, the samples were transferred to a 6-well ultra-low attachment plate, with 12 samples per well. The orbital shaker was maintained at 85 rpm until the end of culture. Expansion medium containing CHIR99021 (Stemcell Technologies, Cat# 72052) was added for the first 3-4 days, and then regular expansion medium (IDM-VA) was used for 32 days, with medium changes every 4 days. In the Matrigel group, hBOs were embedded directly in undiluted Matrigel (Corning, Cat# 356234) at the onset of the expansion stage until the end of the experiment. Finally, maturation medium (IDM+VA) was added, and the hBOs were cultured until the end of culture, with medium changes every 4 days.

The working solution (2 μM SB431542 [Stemcell Technologies, Cat# 72234], 2 μM Noggin [Stemcell Technologies, Cat# 78060.1]) was added throughout the neural induction medium, with 3 μM CHIR99021 supplied during the first 3 days of the Expansion medium and BDNF (Yeasen, Cat# 92129ES25) and GDNF (Stemcell Technologies, Cat# 78058) (both 40 ng/mL) supplemented throughout the maturation medium until the end point.

For ClinoStar culture, organoids were transferred to the ClinoStar bioreactor (CelVivo) after the neural induction stage, with approximately 36 organoids per chamber. The bioreactor was operated at 35 rpm with clockwise rotation. The bioreactor chambers were completely filled with culture medium (10 mL), with an approximate working volume of 10 mL per chamber, to minimize air bubbles and maintain stable rotational culture conditions. Cultures were maintained under 5% O_2_, 5% CO_2_, and 37 °C.

### Computational modeling of shear exposure

To estimate the relative shear exposure under the ClinoStar culture condition, an engineering-scale computational model was generated in COMSOL Multiphysics 6.4. The ClinoStar chamber was approximated as a completely filled annular fluid domain, based on the chamber dimensions reported for the rotating culture system, with an annular height of 8.8 mm and inner and outer radii of 14.05 and 23.65 mm, respectively. The culture medium was modeled as an incompressible Newtonian fluid with water-like properties, using a density of 1000 kg/m³ and a dynamic viscosity of 1.0 × 10^-^³ Pa·s. A representative organoid was modeled as a spherical object with a radius of 0.75 mm positioned within the annular chamber.

Because the ClinoStar chamber is completely filled and rotates as a closed system, the main source of local shear was modeled as the relative slip between the organoid and surrounding medium rather than bulk fluid motion against a stationary organoid. A creeping-flow approximation was used to estimate the organoid surface shear stress under prescribed organoid-medium relative slip velocities of 1, 10, 100, 1000, 10 000, and 50 000 μm/s. For each condition, the average and maximum viscous shear stress on the organoid surface were extracted.

For comparison, a 6-well orbital shaker condition was modeled using a representative 6-well geometry containing 3 mL of medium per well and an orbital shaking speed of 85 rpm. The organoid was modeled as a fixed spherical object in the fluid domain, and the resulting average and maximum organoid surface shear stresses were used as reference values. The simulations were intended to provide an engineering estimate of relative shear exposure rather than direct biophysical measurement of shear stress. Therefore, the results were interpreted as supporting a potential reduction in effective shear exposure under filled, co-rotating ClinoStar conditions, provided that organoid-medium relative motion remains limited.

### Cell viability

Cell viability was assessed using the CCK8 kit (Med Chem Express, Cat# HY-K0301) on mature hBOs. Individual hBOs were transferred into 96-well plates, and 100 μl of the CCK8 working solution was added to each well according to the manufacturer’s instructions. Plates were incubated at 37 °C, and absorbance at 450 nm was measured using a microplate reader. OD values at multiple time points (0.5-64 h) were recorded to evaluate metabolic activity and proliferation.

### Single-cell RNA sequencing data processing and analysis

Day-25 hBOs at the neuroepithelial expansion stage were collected for single-cell RNA sequencing. For library preparation, a total of 96 hBOs from each group were pooled and mechanically dissociated, followed by enzymatic digestion for 30 min according to tissue condition. Digestion was terminated, and the cell suspension was centrifuged, resuspended, and filtered sequentially through 70 μm and 40 μm cell strainers to remove undigested tissue fragments and cell aggregates. The cell pellet was washed twice with 1× PBS containing 0.04% BSA and gently resuspended using wide-bore pipette tips to minimize cell damage. Cell concentration and viability were assessed using an automated cell counter. Approximately 1 × 10^6^ cells were collected for sequencing, and the final single-cell suspension was adjusted to approximately 1000 cells/μL before library preparation.

Single-cell RNA-seq libraries were constructed using the DNBelab C Series High-throughput Single-Cell RNA Library Preparation Kit V3.0 (MGI, #940-001819-00) according to the manufacturer’s instructions. Briefly, single-cell suspensions were used for droplet generation, emulsion disruption, bead collection, reverse transcription, second-strand synthesis, cDNA amplification, and indexed library amplification. Library fragment size distribution was assessed using an Agilent 4200 TapeStation, and library concentration was quantified using a Qubit dsDNA HS assay. Qualified libraries were sequenced on the DNBSEQ-T7 platform at HaploX Biotechnology (Jiangxi, China).

Raw single-cell RNA sequencing data were analyzed using Seurat v4.3 in R. Cells with fewer than 500 detected genes or with mitochondrial transcript content greater than 10% were classified as low-quality and removed. After quality-control filtering, 18 432 high-quality cells were retained for downstream analysis. Data were normalized using SCTransform, followed by principal component analysis. A shared nearest-neighbor graph was constructed using the top principal components, and cell clusters were visualized in 2 dimensions using Uniform Manifold Approximation and Projection (UMAP).

#### UMAP construction and clustering

Single-cell RNA sequencing data from Day 25 (Expansion stage) human hBOs were analyzed using Seurat (v4.3) in R. For library preparation, a total of 48 hBOs from each group were pooled and dissociated into a single-cell suspension. Approximately 1 × 10^6^ cells were collected for sequencing. Cells with fewer than 500 detected genes or with mitochondrial transcript content greater than 10% were classified as low-quality and subsequently removed. After the quality-control screening, 18432 high-quality cells were retained for further analysis. The data were normalized using SCTransform, followed by principal component analysis. Using the top principal components, a shared nearest neighbor graph was built for clustering distinct cell populations, with clusters visualized in 2 dimensions by UMAP.

#### Cell-type annotation and statistical analysis

Cell clusters were annotated based on established marker gene expression profiles and the spatial distribution of these markers within the UMAP embedding. Cell types included dorsal progenitors, radial glia, intermediate progenitor-like cells, immature neurons, early excitatory neurons, and additional minor populations. Cell-type composition was quantified by calculating the proportion of cells assigned to each annotated category. These proportions were visualized using bar plots to summarize the cellular composition of 25-day hBOs.

#### Feature visualization and module score analysis

To visualize the pattern of gene lineage, expression of selected marker genes was projected onto the UMAP embedding using Seurat FeaturePlot. Marker genes were chosen to represent key stages along cortical neurogenesis, including radial glia/progenitor markers (SOX2), intermediate progenitor marker (TBR2), early neuronal markers (NEUROD1, DCX), and cortical neuronal differentiation markers (TBR1, CTIP2, SATB, TUJ1). To assess coordinated transcriptional programs corresponding to distinct developmental states, module scores were calculated using Seurat’s AddModuleScore function. Gene sets were defined a priori accordingly: (1) Radial glia module: SOX2, PAX6, HES1, HES5, VIM, FABP7; (2) Neurogenic/IPC module: TBR2, NEUROG2, HES6, ASCL1; (3) Cortical neuron module: NEUROD1, DCX, TBR1, CTIP2, SATB, TUJ1. Module scores were visualized on the UMAP embedding to examine spatial enrichment patterns of progenitor, neurogenic intermediate, and neuronal differentiation programs.

### RNA sequencing and analysis

Total RNA was extracted from hBOs (96 per group) using TRIzol reagent and chloroform according to the manufacturer’s instructions. RNA quality was assessed using the Nanodrop, and RNA integrity was evaluated using the Agilent Bioanalyzer 4150 system. Paired-end mRNA sequencing libraries were prepared using an mRNA-seq library preparation kit according to the manufacturer’s instructions and sequenced on an Illumina NovaSeq 6000/MGISEQ-T7 platform. For bioinformatic analysis, the gene expression file was imported into R. Differential expression analysis between the indicated groups was performed using DESeq2. Genes with a false discovery rate (FDR) < 0.05 were considered significantly differentially expressed. KEGG pathway enrichment analysis and GSEA were performed in R using the differentially expressed genes or ranked gene lists from the indicated comparisons. Heatmaps were generated in R to visualize representative differentially expressed genes and pathway-associated genes under different culture conditions.

### Immunofluorescence staining

hBOs at different stages were fixed with 4% paraformaldehyde overnight at 4 °C and cryoprotected in 30% sucrose at 4 °C. Then, hBOs were incubated with the gelatin solution (10 g sucrose + 100 mL DPBS + 7.5 g gelatin) at 37 °C for 1 h. A phenol red-containing gelatin solution (100 mL gelatin solution + 0.5 g Phenol red powder) was prepared for tissue embedding. Once the hBOs were positioned in the mold, the phenol red-containing gelatin solution was added. Dry ice was added to 100% ethanol to prepare a dry ice/ethanol slurry. The samples were kept in the cold slurry until completely frozen and stored at -80°C. The hBOs were cryosectioned at 15 μm. The slides were washed with PBST at 37 °C for 10 min to remove the gelatin. Blocking was performed with 5% BSA + 1% Triton DPBS solution for 2 h, and the primary antibody was incubated at 4 °C overnight, followed by the secondary antibody mixture at room temperature for 2 h. Primary antibodies were used at the following dilutions: SOX2 (1:50), TUJ1 (1:100), Ki67 (1:100), SATB (1:50), CTIP2 (1:50), TBR2 (1:50), and TBR1 (1:50). DAPI-containing mounting medium was added to cover the slides, and the slides were stored at 4 °C for 1-2 weeks. The antibody catalog numbers are listed in Table 1.

**Table 1.** Antibody details.

Antibody	Source	Identifier
**Mouse antibody SOX2**	R&D	Cat#MAB2018; RRID: AB_358009
**Rabbit antibody Tubulin beta-III (TUJ1)**	HUABIO	Cat#ET1604-17; RRID: AB_3069688
**Rabbit antibody TBR1**	Abcam	Cat#ab183032; RRID: AB_2936859
**Rat antibody TBR2**	Invitrogen	Cat#14-4875-82; RRID: AB_11042577
**Mouse antibody SATB**	Abcam	Cat#ab51502; RRID: AB_882455
**Rat antibody CTIP2**	Abcam	Cat#ab18465; RRID: AB_2064130
**Rat antibody KI67**	Abcam	Cat#14-5698-82; RRID: AB_10854564
**Goat Anti-Mouse IgG H&L (Alexa Fluor 647) preadsorbed**	Abcam	Cat#ab150119; RRID: AB_2811129
**Donkey Anti-Rabbit IgG H&L (Alexa Fluor 568) preadsorbed**	Abcam	Cat#ab175692; RRID: AB_3076303
**Goat Anti-Rat IgG H&L (Alexa Fluor 488) preadsorbed**	Abcam	Cat#ab150165; RRID: AB_2650997
**Goat Anti-Rabbit IgG H&L (Alexa Fluor 488) preadsorbed**	Abcam	Cat#ab150081; RRID: AB_2734747
**Rabbit antibody RET**	Abcam	Cat#ab134100; RRID: AB_2920824
**Rabbit antibody GFRA1**	Proteintech	Cat#16324-1-AP; RRID: AB_3741883
**Rabbit antibody GAPDH**	HUABIO	Cat# ET1601-4; RRID: AB_3069615
**Goat Anti-Rabbit IgG H&L (HRP)**	Abcam	Cat#ab6721; RRID: AB_955447

### TUNEL staining

Apoptosis was assessed using the TUNEL Apoptosis Detection Kit (YEASEN, Cat#40308ES20) according to the manufacturer’s protocols. Following the reaction, slides were counterstained with DAPI prior to mounting.

### Image analysis

A Nikon Eclipse Ti2-E fluorescent microscope was used for imaging of Immunofluorescent staining and Real-time hBO monitoring. Images were analyzed using ImageJ software (NIH, U.S.). For quantitative analysis, the area and fluorescence intensity of individual hBOs were measured in ImageJ and normalized to the ROI area.

### Overexpression and knockout of GFRA1 in hBOs using lentivirus

GFRA1 knockdown and overexpression lentiviral constructs, together with their corresponding negative controls, were generated using standard molecular cloning methods. Lentiviral particles were produced by co-transfecting HEK293T cells with the shRNA or overexpression vectors together with the lentiviral packaging plasmids pMD2.G, psPAX2, and the transfer plasmid at a ratio of 0.8 μg: 1.2 μg: 2 μg. Viral supernatants were collected at 72 h post-transfection, clarified by centrifugation, and used for subsequent transduction.

For lentiviral transduction, hBOs at Day 40 were collected from the indicated culture groups and incubated with the corresponding viral supernatants. For Matrigel-embedded hBOs, they were first gently dissociated from the surrounding Matrigel by pipetting before viral exposure. After viral incubation, hBOs were returned to fresh culture medium and maintained under their original culture conditions.

### Western blot

For each biological replicate, multiple hBOs from the same experimental group were pooled and lysed in RIPA buffer containing a protease inhibitor cocktail on ice. For Matrigel-embedded hBOs, they were first mechanically dissociated from Matrigel by gentle pipetting before lysis. After centrifugation at 12 000 *g* for 10 min at 4 °C, the supernatants were collected, and protein concentrations were determined using a BCA Protein Assay Kit (Yeasen, Cat# 20200ES76) according to the manufacturer’s instructions. Equal amounts of protein were mixed with loading buffer, denatured at 95 °C for 10 min, separated by SDS–PAGE, and transferred onto PVDF membranes. Membranes were blocked with 5% nonfat milk in TBST for 1 h at room temperature and then incubated with primary antibodies against RET (1:1000), GFRA1 (1:1000), and GAPDH (1:5000) at 4 °C overnight, followed by HRP-conjugated secondary antibodies (1:5000) for 1 h at room temperature. Protein bands were visualized using an enhanced chemiluminescence detection system and imaged with a chemiluminescence imaging system. Band intensities were quantified using ImageJ software and normalized to GAPDH. The antibody catalog numbers are listed in Table 1.

### Quantitative real-time reverse transcription PCR

Total RNA was extracted from hBOs using an RNA extraction kit according to the manufacturer’s instructions. RNA concentration and purity were assessed using a NanoDrop spectrophotometer based on A260/A280 and A260/A230 ratios. cDNA was synthesized from 1 μg of total RNA using a reverse transcription kit (GOONIEBIO, Cat# 500-101) according to the manufacturer’s protocol. Quantitative real-time PCR was performed using SYBR Green Master Mix (GOONIEBIO, Cat# 500-100) on a real-time PCR system. qPCR primers were synthesized by Sigma-Aldrich, and the primer sequences (5′-3′) were as follows: GFRA1, forward GACACCATTGCCCTGAAAGA and reverse TCCGACCCAA CCTGGACTC; RET, forward TCAACCTTCTGAAGACCGGC and reverse CAGCATCAGGCGGTACATCT; CTHRC1, forward GGGCTCATCTGTGGGAAGTATA and reverse TTGAATCCATCCCGACCTGG; GAPDH, forward CTCCGGTGATGCTTTTCCT and reverse GCCCAATACGACCAAATCAGAG. Relative mRNA expression levels were normalized to GAPDH and calculated using the 2^-ΔΔCt^ method.

### Statistical analysis

Statistical analyses were performed using GraphPad Prism (V9, CA, USA). Data are presented as mean ± Standard Deviation (SD). For western blotting and RT-qPCR analyses, “n” represents the number of independent experiments. For comparisons between 2 groups, unpaired 2-tailed Student’s *t*-tests were used. For comparisons among multiple groups, one-way ANOVA with Tukey post hoc tests was used. For comparisons involving 2 independent variables, 2-way ANOVA with Tukey post hoc tests was used. For comparisons involving time-dependent changes, one-way ANOVA with repeated measures was used. *P* < 0.05 was considered statistically significant. For RNA-seq data, differential expression analysis was performed using DESeq2, and genes with a false discovery rate (FDR) < 0.05 were considered significantly differentially expressed.

## Results

### Generation and early cortical characterization of hBOs

**Figure 1.**
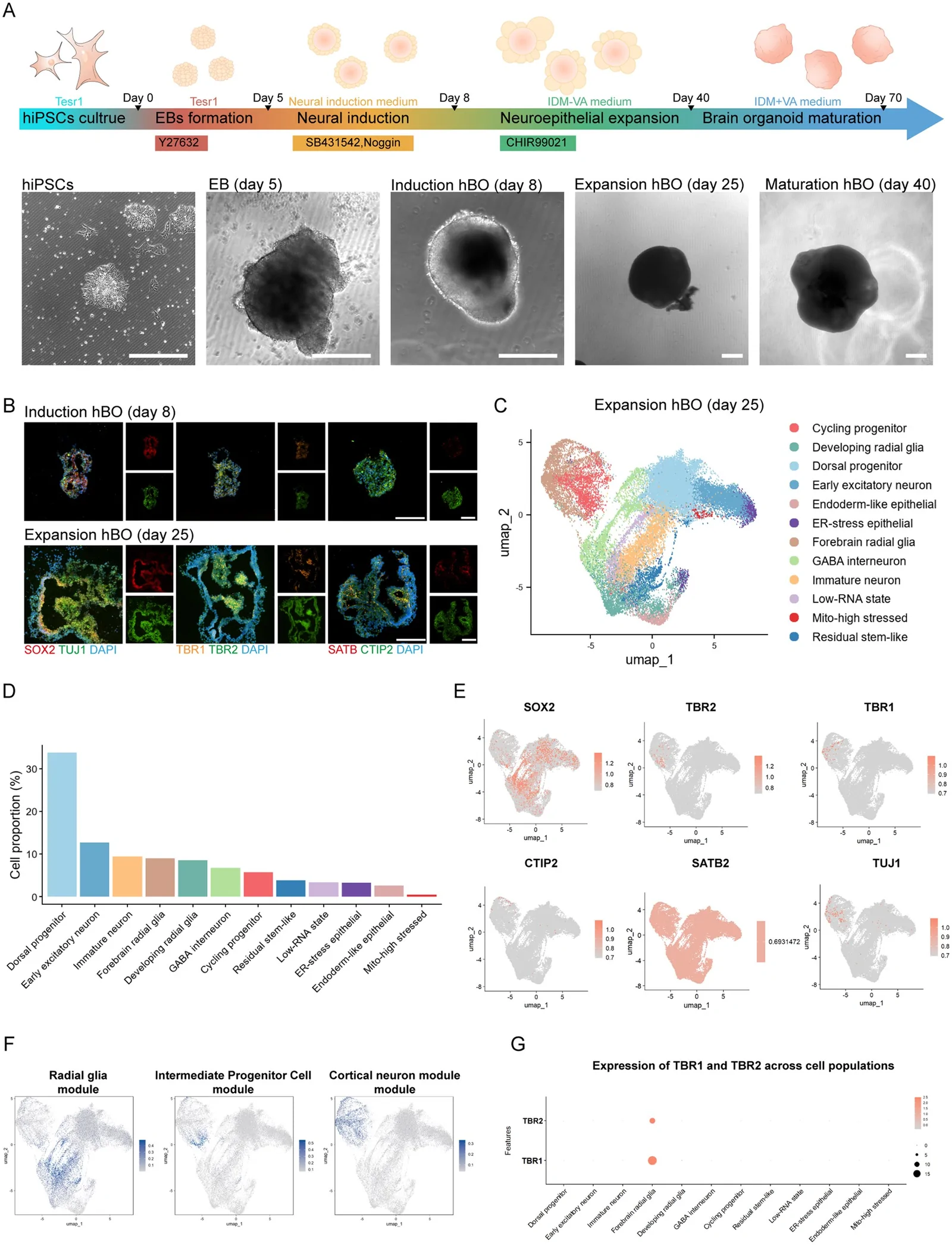
Generation of hBO and single-cell characterization of the neuroepithelial expansion stage.(A) Overview of the differentiation workflow from hiPSCs through embryoid body formation, neural induction stage, neuroepithelial expansion stage, and maturation stage, with representative brightfield images. (B) Immunoflurostaining of SOX2^+^ (ventricular zone–like, neural progenitor cell) rosettes and cortical lineage markers, including TBR2^+^ (subventricular zone–like, Intermediate Progenitor Cell), TBR1^+^ (deep-layer VI neuron), CTIP2^+^ (layer V subcortical projection neuron), SATB^+^ (layer II-IV callosal projection neuron), and TUJ1^+^ (immature neuron) in Induction hBOs at Day 8 and Expansion BO at Day 25. DAPI marks nuclei. Scale bar = 500 μm. (C) UMAP representation of single-cell RNA-sequencing data from 25-day hBOs. Cells are colored by annotated cell types. N=1. (D) Relative proportions of annotated cell types in 25-day hBOs. (E) Feature plots showing the expression of representative SOX2, TBR2, TBR1, SATB, CTIP2, and TUJ1 genes across the UMAP embedding. (F) Module score visualisation of gene sets associated with radial glia, intermediate progenitor cells, and cortical neurons. (G) Dot plot showing the expression patterns of TBR1 and TBR2 across annotated cell populations identified by single-cell RNA sequencing. Dot size represents the percentage of cells expressing the indicated gene within each cluster, and colour intensity indicates the average normalised expression level.

We successfully generated hBOs from human induced pluripotent stem cells (hiPSCs) using a published protocol.^18^ There are 4 developmental stages: embryoid body formation (Days 0-5), neural induction (Days 5-8), neuroepithelial expansion (Days 8-40), and hBO maturation (Days 40-70+) (Figure 1A). Rosettes are neural tube-like structures. During neuroepithelial expansion, hBOs increase in size and form clear SOX2^+^ rosettes (Figure 1B). Surrounding these rosettes, markers of cortical progenitor cells and neurons are arranged in an inside-out manner, suggestive of ventricular zone-like formation and early corticogenesis. This stage marks a key point when hBOs establish basic cortical structure.

To characterize cellular states during the neuroepithelial expansion stage, we performed single-cell RNA sequencing (RNA-seq) of Day 25 hBOs (Expansion stage). Uniform Manifold Approximation and Projection (UMAP) embedding revealed multiple transcriptionally distinct but proximally connected cell populations, consistent with coordinated neural lineage progression rather than discrete, isolated clusters (Figure 1C). Only a small fraction of cells retained residual stem-like features, whereas the majority of cells were assigned to dorsal progenitor, radial glia, intermediate progenitor-like, and early neuronal lineage-associated populations. This composition suggests that the day-25 hBOs had largely acquired neural lineage identity while remaining predominantly within an early neuroepithelial/neurogenic developmental state. (Figure 1D). Visualization of lineage-associated marker genes on the UMAP embedding reflected a continuous transition from radial glia through intermediate progenitors toward differentiating cortical neurons (Figure 1E). SOX2 expression was enriched in progenitor-rich regions, consistent with its association with radial glia/progenitor identity. In contrast, TBR2, TBR1, CTIP2, and TUJ1 were detected in more restricted cell populations, consistent with progressive differentiation toward intermediate progenitor and early neuronal states. SATB showed a broader distribution across the UMAP, likely reflecting its relatively limited expression dynamics at this stage rather than a restricted cluster-specific pattern. Consistent with single-gene patterns, module score analysis further revealed coordinated transcriptional programs corresponding to radial glia, intermediate progenitor, and cortical neuron states (Figure 1F). These modules displayed progressively shifted enrichment across the UMAP, supporting a stepwise transcriptional continuum rather than abrupt state transitions. Dot plot analysis showed that TBR1 and TBR2 expression was low and highly restricted across annotated cell populations, with detectable signals mainly observed in the forebrain radial glia-associated population. These findings indicate limited but detectable early corticogenic marker expression during the Day 25 expansion stage (Figure 1G).

### ClinoStar promotes neuroepithelial integrity and early cortical layer-like programming in hBOs

**Figure 2.**
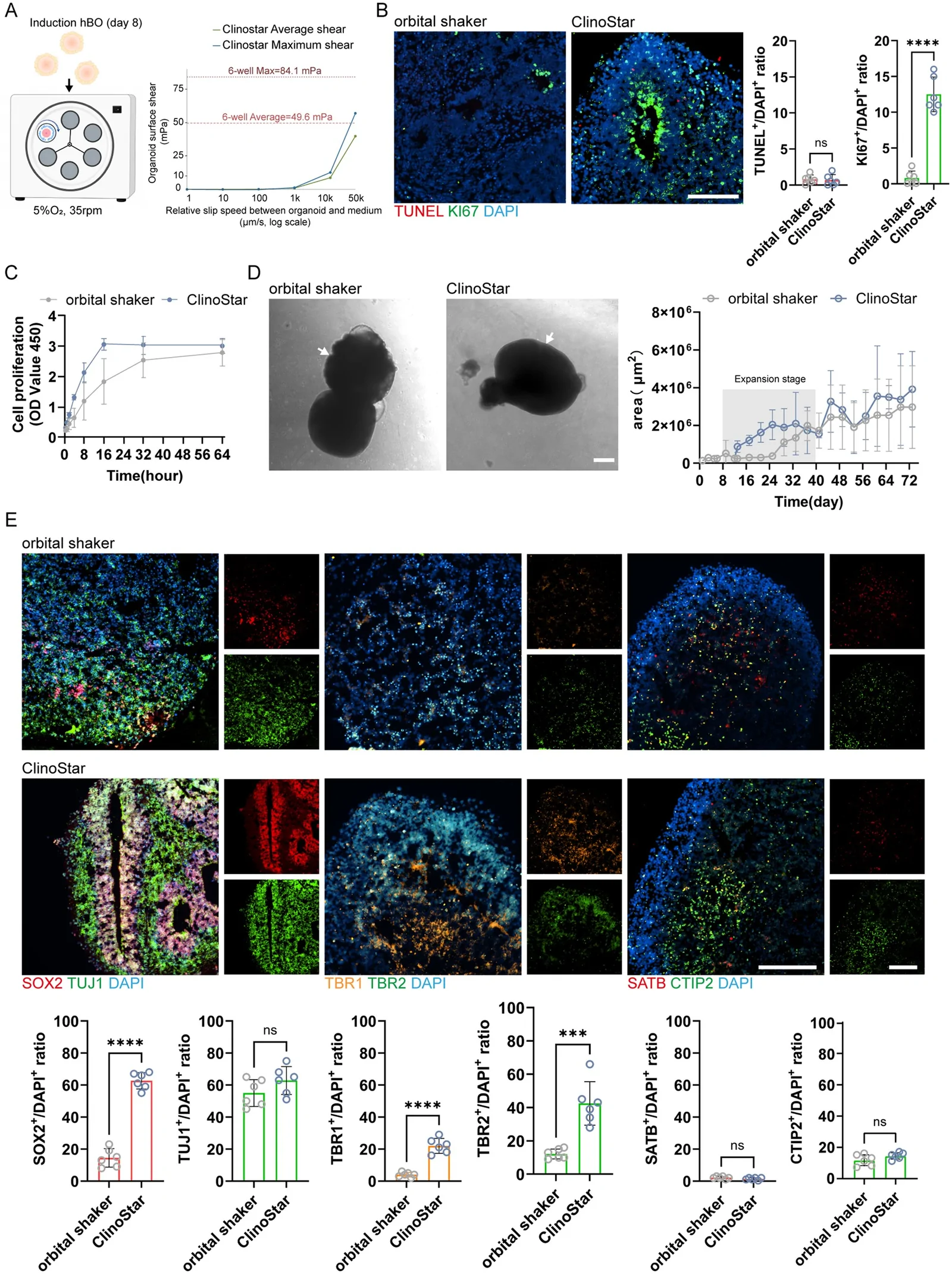
Comparison of hBOs cultured in ClinoStar bioreactors versus orbital shakers. (A) Schematic illustration showing the transfer of 8-day hBOs into the ClinoStar bioreactor for low-shear culture during the neuroepithelial expansion stage, and COMSOL-based estimation of organoid surface shear under ClinoStar conditions compared with a 6-well orbital-shaker reference. (B) Representative images of immunofluorescence staining for TUNEL, Ki67, and DAPI in 40-day hBOs cultured under orbital shaker or ClinoStar conditions. The bar figure represents the ratio of TUNEL-positive and Ki67-positive cells against the total cell number (DAPI+). N = 6. Data are expressed as mean ± SD and were analysed using unpaired 2-tailed Student’s *t*-tests; ns, not significant; ****P < 0.0001. Scale bar = 500 μm. (C) Cell viability of day-40 hBOs cultured under orbital shaker or ClinoStar conditions, measured by CCK-8 assay based on OD450 values over time. N = 6. Data are presented as mean ± SD. (D) Representative brightfield images of 40-day hBO surface morphology in ClinoStar versus orbital shaker culture, and quantification of surface area at different time points. N = 6. Data are expressed as mean ± SD. (E) Representative images of immunofluorescence staining for cortical lineage markers (SOX2, TBR2, TBR1, CTIP2, SATB, and TUJ1) in 40-day hBOs in orbital shaker or ClinoStar culture. The bar figure represents the ratio of fluorescence-positive area against total cell number (DAPI+). N = 6. Data are expressed as mean ± SD and were analysed using unpaired 2-tailed Student’s *t*-tests; ns, not significant; ***P < 0.001; ****P < 0.0001. Scale bar = 500 μm.

Because the neuroepithelial expansion stage is a critical developmental window characterized by rapid organoid growth and neuroepithelial rosette formation, hBOs were transferred to the ClinoStar bioreactor before the onset of this stage. COMSOL-based estimation suggested lower effective organoid surface shear under filled, co-rotating ClinoStar conditions than under the 6-well orbital-shaker reference, supporting the use of this system to assess how low-shear culture conditions affect neuroepithelial organization and early corticogenic marker expression (Figure 2A). At Day 40, ClinoStar-cultured hBOs showed increased Ki67^+^ proliferative cells (Figure 2B), while CCK8 analysis indicated higher overall metabolic activity compared with orbital shaker cultures (Figure 2C). Morphologically, ClinoStar hBOs were smoother, more spherical, and had fewer structural irregularities (Figure 2D). Surface area analysis confirmed faster expansion during neuroepithelial development under ClinoStar conditions.

ClinoStar-cultured hBOs showed improved cortical marker organization compared to those cultured on an orbital shaker. While hBOs in shaker cultures showed loosely arranged neuroepithelial regions and discontinuous SOX2^+^ zones, hBOs in the ClinoStar developed thicker and more compact SOX2^+^ rosettes. Key early corticogenesis markers, TBR2^+^ (marker of subventricular zone-like, intermediate progenitor cell) and TBR1^+^ (marker of deep-layer VI-like neuron), were increased in the ClinoStar group, consistent with improved neuroepithelial-like organization. The number of SATB^+^ (marker of upper-layer cortical neurons) cells rose modestly, and that of CTIP2^+^ (marker of deep-layer cortical neurons) cells was similar between the 2 culture systems (Figure 2E), suggesting the emergence of early-stage cortical neuron subtype programming. Overall, ClinoStar culture promoted better neuroepithelial structure and early cortical layer specification.

### ClinoStar mitigates matrigel-induced structural disruption and restores early cortical specification

**Figure 3.**
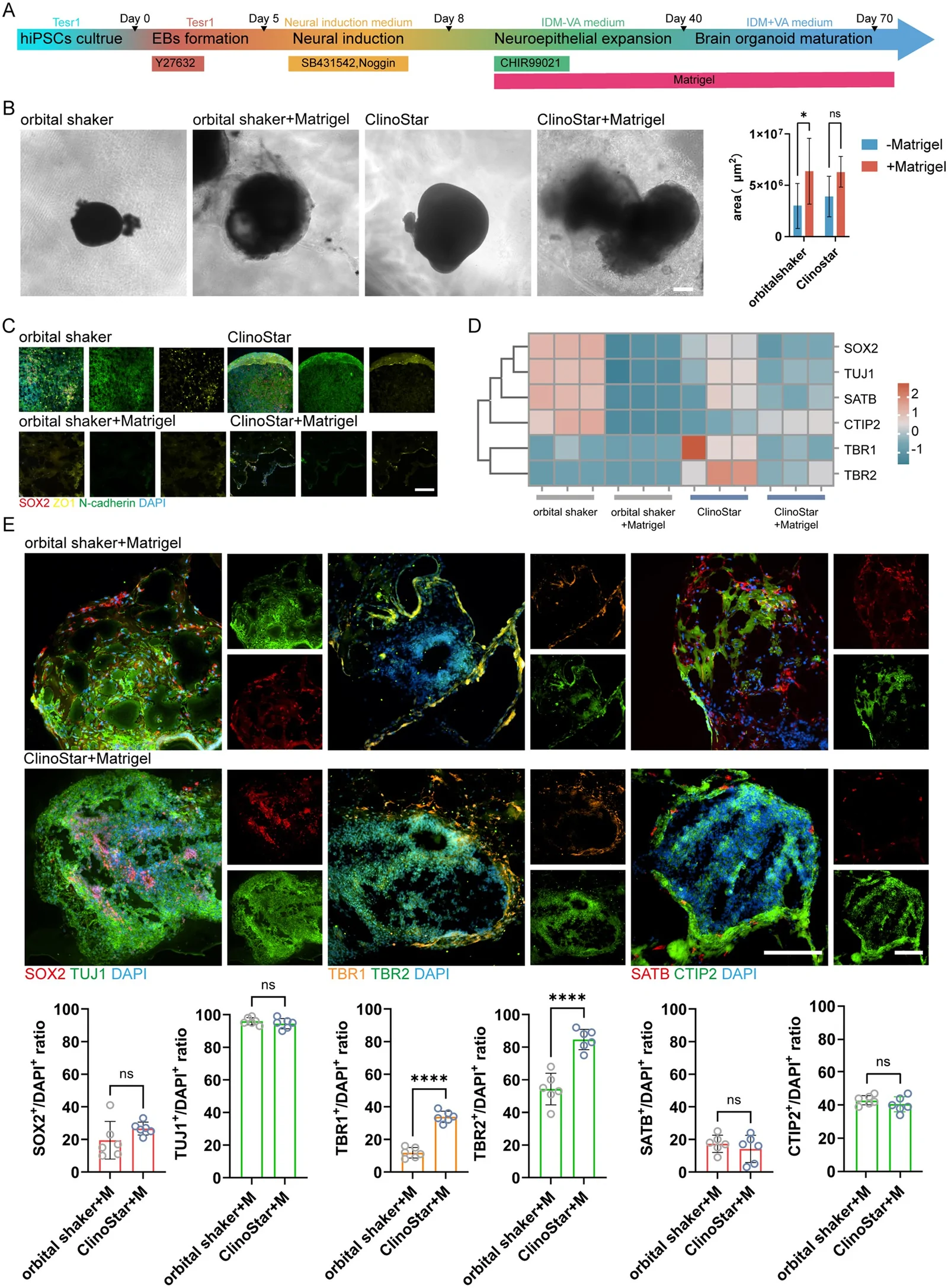
Effects of Matrigel and ClinoStar culture on hBO structure and cortical marker expression. (A) Experimental workflow for evaluating the impact of Matrigel under different culture conditions (orbital shaker vs. ClinoStar). (B) Representative brightfield images of 40-day hBOs in orbital shaker or ClinoStar culture, with or without Matrigel, and quantification of organoid surface area. N = 12. Data are expressed as mean ± SD and were analysed using 2-way ANOVA with post hoc Tukey tests; ns, not significant; *P < 0.05; ****P < 0.0001. Scale bar = 500 μm. (C) Representative images of immunofluorescence staining of SOX2, ZO1, and N-cadherin in 40-day hBOs cultured under orbital shaker or ClinoStar conditions, with or without Matrigel. Scale bar = 500 μm. (D) Heatmap showing patterns of early cortical lineage markers (SOX2, TBR2, TBR1, CTIP2, SATB, and TUJ1) in 40-day hBOs cultured under orbital shaker or ClinoStar conditions, with or without Matrigel. N = 3. (E) Representative images of immunofluorescence staining and quantification of cortical lineage markers (SOX2, TBR2, TBR1, CTIP2, SATB, TUJ1) in 40-day hBOs cultured in ClinoStar system with or without Matrigel. N = 6. Data are expressed as mean ± SD and were analysed using unpaired 2-tailed Student’s *t*-tests; ns, not significant; ****P < 0.0001. Scale bar = 500 μm.

To examine the role of Matrigel in neuroepithelial development, we assessed neuroepithelial development by comparing hBOs with and without Matrigel in both orbital shaker and ClinoStar systems (Figure 3A). Matrigel significantly disrupted neuroepithelial organization during the expansion phase. Under orbital shaker conditions, hBOs embedded in Matrigel developed looser and more fragmented structures with enlarged and irregular morphology, whereas those embedded in Matrigel but cultured in the ClinoStar partially alleviated these structural abnormalities (Figure 3B). Matrigel-embedded hBOs showed less ZO-1 and N-cadherin staining than Matrigel-free hBOs, suggesting Matrigel disrupts junctional organization, weakens cell-cell adhesion, and reduces epithelial polarity (Figure 3C). In the orbital shaker group, Matrigel was associated with reductions in multiple markers in the hBOs, which were less pronounced in hBOs cultured in the ClinoStar system. Among the examined markers, the differences in TBR1 and TBR2 were particularly evident (Figure 3D). ClinoStar culture partly corrected Matrigel-induced hBO developmental defects by restoring TBR2^+^ progenitor aggregation on hBOs surfaces and increasing TBR1^+^ neurons (Figure 3E). Thus, ClinoStar offers mechanical support to stabilize Matrigel-driven disruption in hBO architecture and therefore supports early cortical specification.

### ClinoStar culture is associated with transcriptional enrichment of neuroactive ligand–receptor interaction and cell adhesion programs

**Figure 4.**
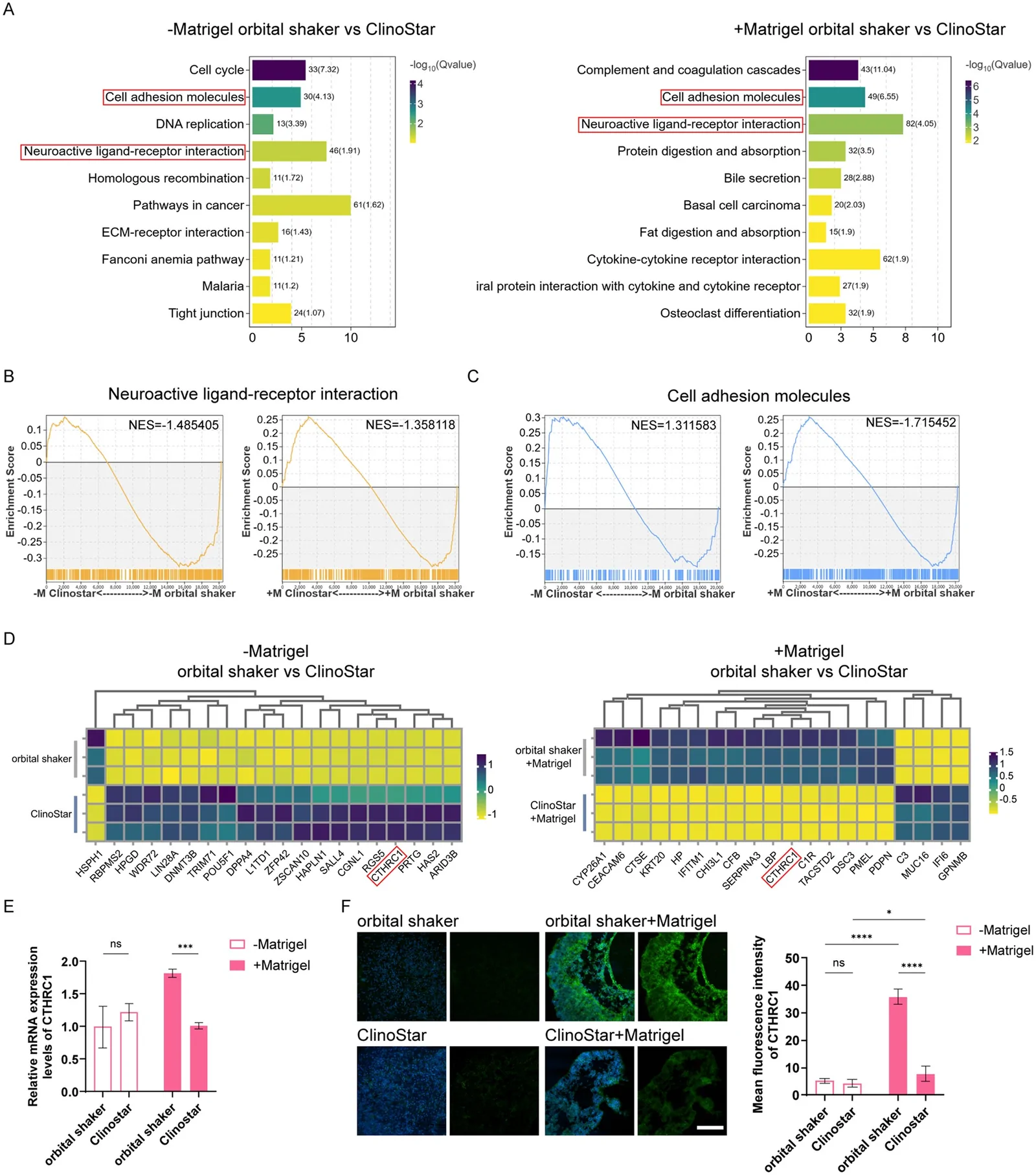
Transcriptomic comparison of hBOs in ClinoStar versus orbital shaker cultures, with or without Matrigel. (A) KEGG pathway enrichment analysis of differentially expressed genes in 40-day hBOs cultured in the orbital shaker vs. ClinoStar system without (left) and with (right) Matrigel. (B) Gene set enrichment analysis (GSEA) of the Neuroactive ligand-receptor interaction pathway in Matrigel-free (left) and Matrigel-containing (right) groups. (C) GSEA plots for the Cell adhesion molecules pathway under Matrigel-free (left) and Matrigel-containing (right) culture conditions. (D) Heatmaps showing representative differentially expressed genes between orbital shaker and ClinoStar cultures under Matrigel-free and Matrigel-treated conditions. CTHRC1 was included among the genes of interest. For CTHRC1, differential expression analysis showed log_2_FC = 2.997, *P* = 9.27 × 10^-24^, FDR = 1.21 × 10^-20^ for the Matrigel-free comparison, and log_2_FC = -4.294, *P* = 8.54 × 10^-76^, FDR = 1.94 × 10^-72^ for the Matrigel-treated comparison. log_2_FC values are shown according to the indicated comparison direction. (E) RT-qPCR analysis showing relative CTHRC1 mRNA expression in 40-day hBOs cultured under orbital shaker or ClinoStar conditions, with or without Matrigel. N = 3. Data are expressed as mean ± SD and were analysed using 2-way ANOVA with post hoc Tukey tests; ns, not significant; ***P < 0.001. (F) Representative images of immunofluorescence staining and quantification of CTHRC1 using one representative field from each organoid in 40-day hBOs cultured under orbital shaker or ClinoStar conditions, with or without Matrigel. N = 6. Data are expressed as mean ± SD and were analysed using 2-way ANOVA with post hoc Tukey tests; ns, not significant; *P < 0.05; ****P < 0.0001. Scale bar = 500 μm.

To explore molecular changes associated with the beneficial effects of ClinoStar culture, we performed transcriptomic analysis comparing hBOs cultured under orbital shaker or ClinoStar conditions, with or without Matrigel (Figure 4A). Pathway enrichment analysis identified neuroactive ligand-receptor interaction and cell adhesion molecules among the major pathways associated with ClinoStar culture, suggesting potential enhancement of extracellular signaling responsiveness and adhesion-related programs. Gene set enrichment analysis (GSEA) showed enrichment of the neuroactive ligand-receptor interaction pathway in hBOs cultured under ClinoStar conditions independent of the presence of Matrigel (Figure 4B). In contrast, enrichment of the cell adhesion molecules pathway was more evident under Matrigel-free conditions and was less pronounced under Matrigel-treated conditions (Figure 4C).

Among the adhesion- and matrix-associated genes, Collagen Triple Helix Repeat Containing 1 (CTHRC1) was particularly notable because its expression pattern separated culture conditions in a matrix-dependent manner (Figure 4D). RT-qPCR confirmed that CTHRC1 was not markedly altered by ClinoStar culture in the absence of Matrigel; however, when Matrigel was present, CTHRC1 was strongly induced under orbital shaker conditions and significantly reduced in ClinoStar-cultured hBOs (Figure 4E). Immunofluorescence staining showed a consistent pattern (Figure 4F). These findings identify CTHRC1 as a sensitive marker of Matrigel-associated ECM remodeling and mechanical stress responses in hBOs, and suggest that ClinoStar low-shear culture attenuates this matrix-induced CTHRC1 response while preserving neuroactive ligand-receptor and adhesion-related transcriptional programmes.

### GDNF supplementation enhances early cortical neuron specification in hBOs

**Figure 5.**
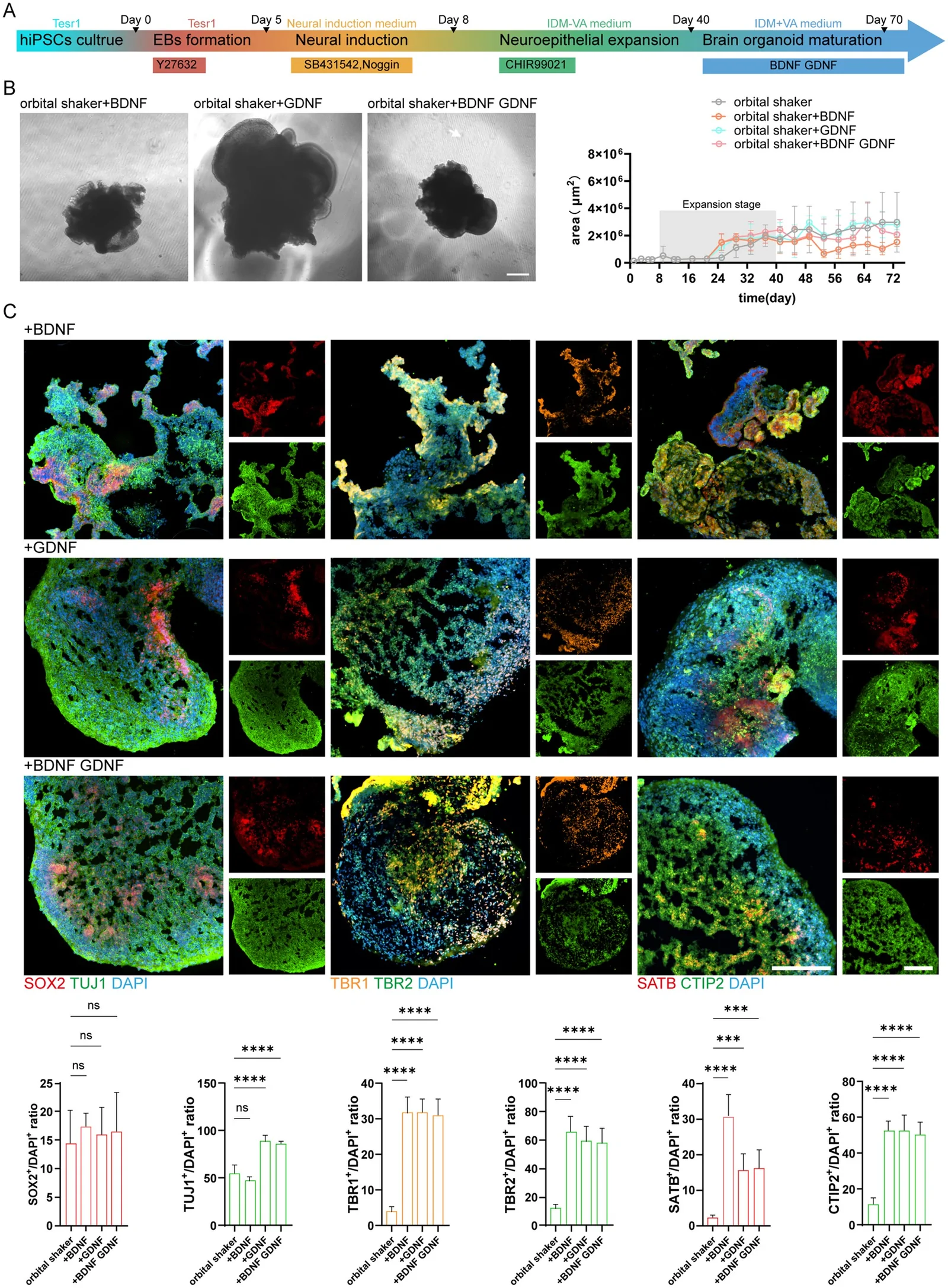
Effects of BDNF and GDNF supplementation on cortical specification in 40-day hBOs.(A) Schematic overview of the culture workflow. (B) Representative images of bright field and quantification of surface area of 40-day hBO with BDNF, GDNF, or BDNF+GDNF supplementations. N = 6. Data are expressed as mean ± SD and were analysed using one-way ANOVA with repeated measures. Scale bar = 500 μm. (C) Representative images of immunofluorescence staining and quantification of 40-day hBOs treated with BDNF, GDNF, or BDNF+GDNF. N = 6. Data are expressed as mean ± SD and were analysed using one-way ANOVA with post hoc Tukey tests; ns, not significant; ***P < 0.001; ****P < 0.0001. Scale bar = 500 μm.

To isolate the contribution of neurotrophic signaling independent of mechanical effects, supplementation experiments were conducted under conventional orbital shaker conditions, allowing the impact of exogenous factors to be evaluated in the absence of low-shear modulation. BDNF and GDNF supplementation (Figure 5A) exerted distinct effects on cortical specification within hBOs. Morphologically, BDNF-treated hBOs appeared to show more peripheral bud-like protrusions, whereas GDNF-treated hBOs showed a tendency toward broader tissue expansion. Longitudinal surface-area analysis did not indicate a clear additive effect in the combined BDNF+GDNF group compared with the single-factor groups (Figure 5B).

Immunofluorescence quantification showed that SOX2^+^ ratios were not significantly changed among the treatment groups, whereas TBR1^+^, TBR2^+^, SATB^+^, and CTIP2^+^ ratios were increased in BDNF- and/or GDNF-treated groups compared with the orbital shaker control (Figure 5C). BDNF, GDNF, and combined BDNF+GDNF treatment produced broadly comparable increases in TBR1^+^ and TBR2^+^ marker-positive populations, while the combined treatment did not show a clear additive effect. These findings suggest that neurotrophic factor supplementation supports early corticogenic marker expression, without indicating a consistent superiority of GDNF over BDNF across the examined markers.

### GFRA1/RET expression is associated with early corticogenic marker patterns under ClinoStar culture

**Figure 6.**
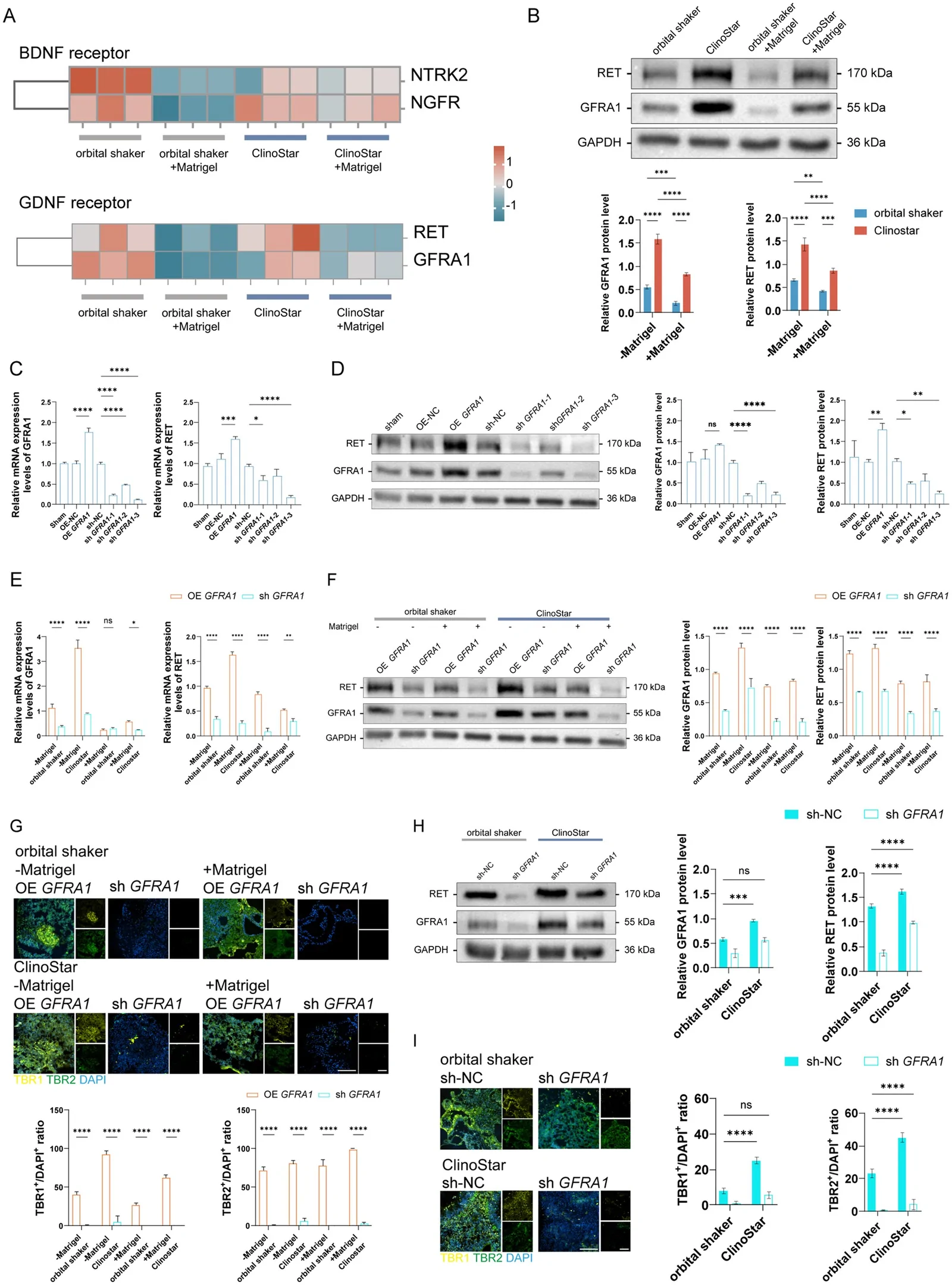
Changes in GDNF receptor signaling and functional modulation of early cortical markers under different culture conditions. (A) Heatmaps of mRNA expression of BDNF receptor-related (NTRK2, NGFR) and GDNF receptor-related (RET, GFRA1) genes derived from bulk RNA-seq analysis of 40-day hBOs cultured under orbital shaker or ClinoStar conditions, with or without Matrigel. (B) Protein levels of RET and GFRA1 in 40-day hBOs cultured under orbital shaker or ClinoStar conditions, with or without Matrigel. GAPDH was used as the loading control. N = 3. Data are expressed as mean ± SD and were analysed using 2-way ANOVA with post hoc Tukey tests; ns, not significant; **P < 0.01; ***P < 0.001; ****P < 0.0001. (C) mRNA expression and (D) protein levels of GFRA1 and RET in 25-day hBOs in the Sham, Overexpression control (OE-NC), GFRA1 overexpression (OE *GFRA1*), Knockdown control (sh-NC), and 3 independent GFRA1 knockdown groups (sh *GFRA1*-1, sh *GFRA1*-2, sh *GFRA1*-3). N = 3. Data are expressed as mean ± SD and were analysed using one-way ANOVA with post hoc Tukey tests; *P < 0.05; ***P < 0.001; ****P < 0.0001. (E) mRNA expression and (F) protein levels of GFRA1 and RET following GFRA1 overexpression (OE *GFRA1*) or knockdown (sh *GFRA1*) under orbital shaker or ClinoStar culture conditions in 40-day hBOs, with or without Matrigel. N = 3. Data are expressed as mean ± SD and were analysed using unpaired Student’s *t* tests between OE and sh treatment; ns, not significant; *P < 0.05; **P < 0.01; ****P < 0.0001. (G) Representative images and quantification of immunofluorescence staining of TBR1, TBR2, and DAPI following GFRA1 overexpression (OE *GFRA1*) or knockdown (sh *GFRA1*) in 40-day hBOs, under orbital shaker or ClinoStar conditions, with or without Matrigel. N = 3. Data are expressed as mean ± SD and were analysed using unpaired Student’s *t* tests between OE and sh treatment; ****P < 0.0001. Scale bar = 500 μm. (H) Protein levels of RET and GFRA1 in 40-day hBOs cultured under orbital shaker or ClinoStar conditions, sh-NC or sh *GFRA1*-3. GAPDH was used as the loading control. N = 3. Data are expressed as mean ± SD and were analysed using 2-way ANOVA with post hoc Tukey tests; ns, not significant; ***P < 0.001; ****P < 0.0001. (I) Representative images and quantification of immunofluorescence staining of TBR1, TBR2, and DAPI following sh-NC and sh *GFRA1-3* in 40-day hBOs, under orbital shaker or ClinoStar conditions. N = 3. Data are expressed as mean ± SD and were analysed using 2-way ANOVA with post hoc Tukey tests; ns, not significant; ****P < 0.0001. Scale bar = 500 μm.

We first examined the expression of neurotrophic factor receptor-associated genes. Heatmap analysis showed that the expression of BDNF receptor-related genes (NTRK2, NGFR) did not changes, whereas the expression of GDNF receptor-related genes (RET, GFRA1) was upregulated in hBOs in the ClinoStar culture system, more so in those without Matrigel support (Figure 6A). The mRNA expression pattern was confirmed by their protein levels measured by Western blot (Figure 6B).

To assess the relevance of GFRA1/RET signaling, we performed lentiviral GFRA1 overexpression and knockdown in day-25 hBOs. qPCR and western blot analyses confirmed effective modulation of GFRA1 expression, with corresponding changes in RET levels, and sh-3 was selected for subsequent experiments because it showed the highest knockdown efficiency (Figure 6C, D). We then examined GFRA1/RET protein expression and TBR1/TBR2 immunofluorescence in day-40 hBOs under different culture and Matrigel conditions (Figure 6E-G). GFRA1 overexpression was accompanied by higher GFRA1/RET expression and more evident TBR1/TBR2 staining, whereas GFRA1 knockdown showed the opposite pattern across the examined conditions. These findings show that GFRA1/RET expression varied with GFRA1 manipulation and was accompanied by corresponding changes in TBR1/TBR2 staining patterns under the tested culture conditions. Further comparison between shNC and shGFRA1 groups showed that, after GFRA1 knockdown, ClinoStar-cultured hBOs retained detectable GFRA1/RET expression and TBR1/TBR2 staining signals, with several readouts approaching the orbital shaker shNC baseline (Figure 6H and I). These findings suggest that ClinoStar culture partially preserves GFRA1/RET-associated receptor expression and TBR1/TBR2-related early corticogenic marker patterns after GFRA1 knockdown. Together, these results support GDNF-GFRA1/RET as a shear stress-associated pathway that may contribute to the maintenance of TBR1/TBR2-related early corticogenic marker patterns in hBOs.

## Discussion

A major limitation in current hBO models is the inconsistent recapitulation of early cortical fate specification, which is highly sensitive to culture-associated mechanical conditions and ECM variability, yet the interplay between these factors and neurotrophic signaling remains poorly defined. In this study, we show that reducing fluid shear using the ClinoStar system is associated with improved neuroepithelial organization and enhanced early cortical differentiation, reflected by increased populations of TBR2^+^ intermediate progenitor- and TBR1^+^ early neuron-associated cells. We further identify that these effects occur alongside enrichment of neuroactive ligand-receptor and cell adhesion-related transcriptional programmes, with increased GFRA1/RET-related GDNF receptor signaling, and that manipulation of GFRA1 is associated with corresponding changes in TBR2/TBR1-associated phenotypes. Together, these findings support a model in which reduced mechanical shear provides a favorable environment for early cortical lineage progression, while highlighting a potential link between mechanical cues and the response of GDNF receptor-related signaling pathways in shaping early corticogenic outcomes in human hBOs.

Reproducing early corticogenesis in human hBOs with high structural accuracy remains challenging. In this study, we identify mechanical culture conditions, ECM-associated microenvironment, and neurotrophic responsiveness as key regulators to determine this early cortical fate in human hBOs. The cellular architecture within hBOs is highly heterogeneous.^1^^,^  ^17^ However, a distinct 6-layer cortical organization is rarely observed. Typically, a hBO contains multiple neuroepithelial rosettes that frequently interfere with one another through fusion events or uneven expansion due to mutual compression. Even when single rosettes were isolated, previous studies often report incomplete or weakly defined laminar patterning.^19^^,^^20^ At the molecular level, the lineage progression from radial glia to intermediate progenitor cells and deep-layer neurons is governed by a tightly regulated transcriptional cascade. PAX6, TBR2, and TBR1 form a sequential hierarchy with reciprocal repression,^21^ establishing a connection between intermediate progenitor cells and deep-layer cortical neurons. TBR2 and TBR1 exhibit mutually exclusive expression patterns,^22^ indicating that the transition between the 2 populations represents a consumption-type lineage step rather than a stable coexistence. Because this transition requires precise spatial organization and balanced progenitor dynamics, any disturbance in mechanical forces, ECM composition, or tissue architecture can prematurely deplete intermediate progenitor cells and impair the generation of early deep-layer neurons,^23^ affecting the fidelity of hBOs in representing the earliest cortical lineage transition. In this context, we found that reducing culture-associated mechanical shear can improve the robustness of early cortical lineage progression. Neuroepithelial organization depends on coordinated cell-cell adhesion and junctional patterning.^24^ Mechanical or ECM-associated perturbations can disrupt epithelial architecture and thereby affect the spatial organization of progenitor and neuronal populations. In this study, ClinoStar-cultured hBOs displayed more organized SOX2^+^ rosette-like structures and relatively continuous ZO-1/N-cadherin-associated boundary patterns, whereas Matrigel-treated hBOs showed less apparent junction-associated staining. These observations suggest that reduced-shear culture may help maintain neuroepithelial-like organization, although more detailed spatial quantification will be required to fully define apical-basal polarity and rosette architecture.

ECM-related mechanics are increasingly recognized as regulators of epithelial cohesion, polarity, and epithelial–mesenchymal transition-like programs.^25^^,^^26^ In our system, Matrigel embedding introduced additional structural perturbations, leading to loosening of neuroepithelial rosettes, less apparent ZO-1/N-cadherin-associated junctional patterns, and suppression of early cortical marker expression. In parallel, transcriptomic analysis revealed enrichment of cell adhesion-related programs under ClinoStar culture, with CTHRC1 emerging as a matrix-responsive ECM-associated gene.^27^^,^^28^ CTHRC1 levels were changed in a matrix-dependent manner, increased under orbital shaker + Matrigel conditions and reduced in ClinoStar + Matrigel hBOs. Previous studies have linked CTHRC1-positive stromal states to mechanically responsive tissue remodeling^29^ and reported a role for CTHRC1 in stem-cell differentiation through PDZ-binding motif (TAZ)-related regulation.^30^ Together, these findings suggest that ECM and culture platform-associated mechanical conditions interactively influence adhesion- and ECM-related molecular states during early hBO development. However, it remains to be determined whether CTHRC1 is directly regulated by BDNF or GDNF, or whether it directly controls neuroepithelial cohesion rather than reflecting matrix-associated remodeling.

The role of neurotrophic signaling was further supported by the differential effects of BDNF and GDNF supplementation. In this study, GDNF showed a stronger effect than BDNF in increasing TBR2^+^ and TBR1^+^ cell populations in the ClinoStar culture system, consistent with the enrichment of neuroactive ligand–receptor interaction pathways during RNA-seq analysis. BDNF-treated hBOs developed prominent peripheral neural bud–like protrusions, a morphology often associated with increased neuronal output and tangential expansion of neuroepithelial domains. This suggests that BDNF not only promotes laminar fate specification but may also stimulate localized growth and outward neurogenic budding, reflecting a broader pro-neurogenic influence on early cortical tissue organisation.^31^^,^^32^ Although crosstalk between neurotrophic and TGF-β-related pathways has been reported in other systems,^33^^,^^34^ our findings do not directly address the underlying signaling mechanisms.^35^ GFRA1/RET was elevated under reduced-shear conditions, and GFRA1 overexpression or knockdown affected TBR1/TBR2-associated phenotypes across different culture conditions. These findings do not exclude contributions from additional neurotrophic or mechanosensitive pathways, but they identify GFRA1/RET signaling as a key association with the improved early corticogenic phenotype observed under ClinoStar culture. Collectively, these findings are consistent with the possibility that growth factor responsiveness contributes to progenitor-to-neuron transitions during early cortical development in hBOs.

We acknowledge that this study is limited by the use of a single hiPSC line. As variability between lines may influence hBOs development and responsiveness to culture conditions, replication in additional independent hiPSC lines will be important to confirm the general applicability of our findings and will be pursued in future revisions. In addition, several other limitations should be considered. First, our analysis primarily focused on early cortical fate specification and did not fully address later stages of neuronal maturation or functional integration. Second, although we observed changes in marker expression and hBO morphology, quantitative spatial analysis of neuroepithelial architecture and apical-basal polarity was limited. Therefore, more advanced imaging and computational approaches may be needed for comprehensive structural assessment. Third, the study relied on supplementation with exogenous factors (BDNF and GDNF) in conventional orbital shaker conditions, which may not entirely recapitulate endogenous signaling dynamics present in vivo, which warrants investigation in future studies.

## Conclusions

In summary, this study demonstrates that early cortical fate specification in human hBOs is highly sensitive to culture-associated mechanical shear stress and ECM conditions. ClinoStar-associated low-shear culture environment improved neuroepithelial-like organization, partially mitigated Matrigel-induced structural disruption, and supported the expression of TBR2^+^/TBR1^+^-associated early cortical differentiation markers, which may be closely associated with the GDNF receptor signaling pathway. This work provides practical guidance for improving the reproducibility and structural fidelity of early cortical hBO models.

## Data Availability

The data underlying this article are available from the corresponding author upon reasonable request.
